# A multifunctional ultrathin flexible bianisotropic metasurface with miniaturized cell size

**DOI:** 10.1038/s41598-021-97930-z

**Published:** 2021-09-16

**Authors:** Tania Tamoor, Nosherwan Shoaib, Fahad Ahmed, Tayyab Hassan, Abdul Quddious, Symeon Nikolaou, Akram Alomainy, Muhammad Ali Imran, Qammer H. Abbasi

**Affiliations:** 1 National University of Sciences and Technology (NUST), Research Institute for Microwave and Millimeter-Wave Studies (RIMMS), Islamabad, 44000 Pakistan; 2 University of Cyprus, KIOS Research and Innovation Center of Excellence, Nicosia, 2109 Cyprus; 3 Frederick Research Center (FRC) and Frederick University, 1036 Nicosia, Cyprus; 4 Queen Mary University of London, School of Electronic Engineering and Computer Science, E1 4NS London, UK; 5 University of Glasgow, James Watt School of Engineering, Glasgow, G12 8QQ UK; 6 Artificial Intelligence Research Center (AIRC), Ajman University, Ajman, 346 UAE

**Keywords:** Electrical and electronic engineering, Electronic and spintronic devices

## Abstract

In this paper, a flexible bianisotropic metasurface possessing omega-type coupling is presented. The designed metasurface behaves differently when excited from either forward (port 1) or back (port 2) sides. It provides an absorption of 99.46% at 15.1 Gigahertz (GHz), when illuminated from port 1, whereas, on simultaneous illumination from port 2, it behaves like a partially reflective surface (PRS). Furthermore, the presented metasurface not only acts as an in-band absorptive surface (port 1) and partially reflective surface (port 2), but it also provides 97% out-of-band transmission at 7.8 GHz. The response of the presented metasurface remains the same for both transverse Electric (TE) and transverse magnetic (TM) polarized wave or any arbitrary linearly polarized wave. Additionally, the response of the metasurface is angularly stable for any oblique incidence up to 45º. The proposed ultrathin flexible metasurface with absorption, partial reflection and out-of-band transmission properties can be used in the Fabry Perrot cavity antenna for gain enhancement with radar cross-section (RCS) reduction both for passband and stop-band filtering, and conformal antenna applications.

## Introduction

Electromagnetic (EM) metamaterials are artificially engineered EM-structures comprising of sub-wavelength periodic i.e., dielectric or metallic structures that resonantly couple to the EM fields while exhibiting properties that are not readily found in nature^1^. As a successor of metamaterials, two-dimensional metasurfaces^2^, owing to their ease of fabrication, have attracted a lot of attention for producing nearly arbitrary wavefront and phase shift of EM waves. Due to their rare and exceptional abilities to distort phase, polarization and the amplitude of EM waves, they play a key role in antenna performance enhancement, superlenses, scattering reduction,phase manipulation, reflection control, or polarization rotation^3–10^. Another interesting area in this emerging field is the absorption of EM waves via anisotropic structures. Several such designs have been reported in the literature^11–14^, but they usually perform with a single functionality due to the incorporation of a complete ground plane. The current research impetus is not only to design compact but also multifunctional absorptive structures. The exploitation of the opportunity to design a resonant absorber, which is transparent outside of the absorption band, could open up various novel prospects in applications, for instance, in ultrathin filters for wave trapping, selective multifrequency bolometers, and sensors^15^. To attain multiple functionalities from a single metasurface, the following two techniques can be possibly used:

1. Multi-layered metasurface
2. Bianisotropic metasurface

Although, a multilayer metasurface has the advantage of wider bandwidth, it exhibits some disadvantages including fabrication complexity, high cost, incident angle dependency and bulky size^16^. The only suitable, cost-effective and unexplored technique to achieve multiple functionalities in absorptive metasurfaces is bianisotropy^18–27^.

Metasurfaces that exhibit electromagnetic coupling are known as bianisotropic metasurfaces. Bianisotropic metasurfaces can be classified into four categories depending on their functionalities and internal properties. In^28^, the classification of bianisotropic materials has been discussed based on the magneto-electric and electromagnetic dyadic nature for both reciprocal media (i.e., chiral and omega) and non-reciprocal media (i.e., Tellegen and moving particle). By enabling Chiral coupling and Tellegen coupling in a metasurface, one may control the reciprocal and non-reciprocal polarization transformation. Similarly, asymmetric co-polarized reflection and transmission can be achieved through Omega and artificial moving particle coupling. Taking this into consideration, Ra’di et al.^29^ discussed the theoretical modeling of omega bianisotropic metasurfaces and the design possibilities through reciprocal omega coupling. One of the key functionality that omega coupling enables in a structure is the asymmetric absorption. To attain perfect absorption when illuminated from one side and the multiple functionalities when the same metasurface is illuminated from the other side, both uniaxial reciprocal (omega) and non-reciprocal (Tellegen and AM) bianisotropic properties, are required. The research community mainly focus on reciprocal metasurfaces due to their ease of fabrication, low cost, and lack of active elements (i.e. transistors and diodes) in the structure.

Based on the theoretical analysis given by Ra’di et al^29^, the Yzadi et al^30^ proposed a simple reciprocal bianisotropic metasurface consisting of metallic square patches that possess omega coupling for the very first time. The designed structure provides perfect absorption when illuminated from one side and gives asymmetric reflection when illuminated from the other side. However, its claimed performance deviates when exposed to different polarized waves (polarization between TE and TM). In addition, it provides very low out-of-band transmission of 20%. Recently in order to overcome this limitation, Fahad et al^31,32^. proposed a metasurface exhibiting polarization-insensitive behavior. Although the polarization insensitivity problem was addressed, it came with a toll of having a larger unit-cell size and low out-of-band transmission value of 10%^31,32^. The low out-of-band transmission and larger unit-cell size limit the usage of these designs for several applications.

In this paper, a miniaturized ultra-thin flexible bianisotropic metasurface is designed and discussed. The proposed metasurface is not only flexible but it also provides more than 97% out-of-band transmission power, thus, overcoming the transmission-based limitations. The maximum out-of-band transmission is achieved at 7.8 GHz, thus, enabling the band-pass filtering functionality, which is a salient feature of the proposed metasurface. Maximum absorption of 99.4% at 15.1 GHz when illuninated from port 1 and partial reflections of nearly 99% when illuminated from port 2 are achieved through the same structure. The maximum out-of-band transmission with bandpass filtering functionality, the miniaturized size, and the flexible substrate make the proposed design a suitable prospect for several applications.

## Theoretical analysis and design evolution

### Theoretical background

It is evident from^28^ literature that to achieve perfect absorption, the thickness of a metasurface cannot be zero, as a result of the existence of at least two induced moments e.g., the electric and the magnetic moment. These induced dipole moments are linearly dependent on the externally applied incident field and can be related using polarizability dyadic as follows:

1 $$\left[ {\begin{array}{*{20}c} p \\ m \\ \end{array} } \right] = \left[ {\begin{array}{*{20}c} {\overline{\overline{{\hat{\alpha }}}}_{ee} } & {\overline{\overline{{\hat{\alpha }}}}_{em} } \\ {\overline{\overline{{\hat{\alpha }}}}_{me} } & {\overline{\overline{{\hat{\alpha }}}}_{mm} } \\ \end{array} } \right]*\left[ {\begin{array}{*{20}c} {E_{inc} } \\ {H_{inc} } \\ \end{array} } \right]$$

here $E_{inc}$ is an incident electric field and $H_{inc}$ is an incident magnetic field, which are related as follows:

2 $$H_{inc} = 1 \pm { }\frac{1}{{_{0} }}\overline{\overline{J}}_{t} E_{inc}$$

In Eq. 1, $\overline{\overline{{\hat{\alpha }}}}_{ee} ,\overline{\overline{{\hat{\alpha }}}}_{mm } ,\overline{\overline{{\hat{\alpha }}}}_{em }$ and $\overline{\overline{{\hat{\alpha }}}}_{me}$ are the effective electric, magnetic, electro-magneto, and magneto-electric polarizability components of the proposed metasurface. The effective polarizabilities include the effects of a single particle in an array^29^. The terms $\overline{\overline{{\hat{\alpha }}}}_{ee}$,$\overline{\overline{{\hat{\alpha }}}}_{mm}$ show the electric response to an incident electric field and the magnetic response to an incident magnetic field, respectively. The other two terms $\overline{\overline{{\hat{\alpha }}}}_{me}$ and $\overline{\overline{{\hat{\alpha }}}}_{em}$ represent bianisotropic coupling dyadics. Due to uniaxial symmetry, only isotropic response and rotation around $z_{o}$ axis is allowed, hence polarizability dyadics can be written as:

3 $$\overline{\overline{{\hat{\alpha }}}}_{ee} = \hat{\alpha }_{ee}^{co} \overline{\overline{I}}_{t} + \hat{\alpha }_{ee}^{cr} \overline{\overline{J}}_{t}$$

4 $$\overline{\overline{{\hat{\alpha }}}}_{em} = \hat{\alpha }_{em}^{co} \overline{\overline{I}}_{t} + \hat{\alpha }_{em}^{cr} \overline{\overline{J}}_{t}$$

5 $$\overline{\overline{{\hat{\alpha }}}}_{me} = \hat{\alpha }_{me}^{co} \overline{\overline{I}}_{t} + \hat{\alpha }_{me}^{cr} \overline{\overline{J}}_{t}$$

6 $$\overline{\overline{{\hat{\alpha }}}}_{mm} = \hat{\alpha }_{mm}^{co} \overline{\overline{I}}_{t} + \hat{\alpha }_{mm}^{cr} \overline{\overline{J}}_{t}$$

Here

7 $$\overline{\overline{I}}_{t} = \overline{\overline{I}} - z_{O} z_{O}$$

8 $$\overline{\overline{J}}_{t} = \overline{\overline{I}} - z_{O} *\overline{\overline{I}}_{t}$$

where $c_{o}$ and $c_{r}$ indicate the symmetric and antisymmetric parts of polarizability dyadic, respectively. $\overline{\overline{I}}_{t}$ represent transverse unit dyadics and $\overline{\overline{J}}_{t}$ represent a vector product operator. Thus, dipole moments can also be written as:

9 $$\left[ {\begin{array}{*{20}c} p \\ m \\ \end{array} } \right] = \left[ {\begin{array}{*{20}c} {\overline{\overline{{\hat{\alpha }}}}_{ee} \pm { }\frac{1}{{\eta_{o} }}{ }\overline{\overline{{\hat{\alpha }}}}_{{em{ }}} \left( {z_{o} \overline{\overline{I}}_{t} { }} \right)} \\ {\overline{\overline{{\hat{\alpha }}}}_{me} \pm { }\frac{1}{{\eta_{o} }}{ }\overline{\overline{{\hat{\alpha }}}}_{{me{ }}} \left( {z_{o} \overline{\overline{I}}_{t} { }} \right)} \\ \end{array} { }} \right] \cdot E_{inc}$$

Solving the above equation while assuming an infinite sheet of electric and magnetic currents, the following expressions for the reflected and transmitted electric fields may be obtained^33^:

10 $$E_{r} = - \frac{j\omega }{{2S}}\left\{ {\begin{array}{*{20}c} {\left[ {\left. {\eta_{0} \hat{\alpha }_{ee}^{co} \pm \hat{\alpha }_{em}^{cr} \pm \hat{\alpha }_{me}^{cr} - \frac{1}{{\eta_{0} }}\hat{\alpha }_{mm}^{co} } \right]\overline{\overline{I}}_{t} } \right. + } \\ {\left[ {\left. {\eta_{0} \hat{\alpha }_{ee}^{cr} \mp \hat{\alpha }_{em}^{co} \mp \hat{\alpha }_{me}^{co} - \frac{1}{{\eta_{0} }}\hat{\alpha }_{mm}^{cr} } \right]} \right.\overline{\overline{J}}_{t} } \\ \end{array} } \right\} \cdot E_{inc}$$

11 $$E_{t} = \left\{ {\begin{array}{*{20}c} {\left[ {1 - \frac{j\omega }{{2S}}\left( {\eta_{0} \hat{\alpha }_{ee}^{co} \pm \hat{\alpha }_{em}^{cr} \mp \hat{\alpha }_{me}^{cr} + \frac{1}{{\eta_{0} }}\hat{\alpha }_{mm}^{co} } \right)} \right]\overline{\overline{I}}_{t} - } \\ {\frac{j\omega }{{2S}}\left[ {\left. {\eta_{0} \hat{\alpha }_{ee}^{cr} \mp \hat{\alpha }_{em}^{co} \pm \hat{\alpha }_{me}^{co} + \frac{1}{{\eta_{0} }}\hat{\alpha }_{mm}^{cr} } \right]} \right.\overline{\overline{J}}_{t} } \\ \end{array} } \right\} \cdot E_{inc}$$

It can be seen from the above equations that the presence of cross-coupling dyadic causes the asymmetry in the interaction of the incident plane waves. Further modifying Eqs. 10 and 11, for asymmetric absorption, the reflected $E_{r}$ and transmitted $E_{t}$ electric fields can be written as^29^:

12 $$E_{r} = \frac{j\omega }{S}\left\{ { \pm \left[ {\left. {\hat{\alpha }_{em}^{cr} + \hat{\alpha }_{me}^{cr} } \right]\overline{\overline{I}}_{t} } \right. \mp \left[ {\left. {\hat{\alpha }_{em}^{co} \mp \hat{\alpha }_{me}^{co} } \right]} \right.\overline{\overline{J}}_{t} } \right\} \cdot E_{inc}$$

13 $$E_{t} = \frac{j\omega }{S}\left\{ { \pm \left[ {\left. {\hat{\alpha }_{em}^{cr} - \hat{\alpha }_{me}^{cr} } \right]\overline{\overline{I}}_{t} } \right. \mp \left[ {\left. {\hat{\alpha }_{em}^{co} - \hat{\alpha }_{me}^{co} } \right]} \right.\overline{\overline{J}}_{t} } \right\} \cdot E_{inc}$$

It is evident from the above relationship that by tuning one side of the structure as a perfect absorber, one may achieve special properties like asymmetric reflection and transmission from the other side. Reciprocal structures that enable such behavior are known as omega structures. They possess omega-type coupling that is responsible for enabling asymmetric reflections and perfect absorption simultaneously in the same metasurface. For the reciprocal structure, electric and magnetic polarizability dyadic are symmetric i.e., $\alpha_{ee}^{cr}$, $\alpha_{mm}^{cr} = 0$, while the bianisotropic coupling dyadic meets the following conditions:

14 $$\hat{\alpha }_{em}^{co} = - \hat{\alpha }_{me}^{co}$$

15 $$\hat{\alpha }_{em}^{cr} = \widehat{ \alpha }_{me}^{cr}$$

where $\alpha_{em}^{cr}$ represents omega coupling dyadic and ${ }\alpha_{em}^{co}$ represents chiral coupling dyadic. For an ultimately thin symmetric absorber, the electric and magnetic polarizabilities should be in Huygens balance^30^:

16 $$\hat{\alpha }_{mm} = \eta^{2} \hat{\alpha }_{ee}$$

Furthermore, asymmetric absorption/reflection from any structure can be realized when the following conditions are satisfied^29^

17 $$\hat{\alpha }_{mm} = \eta \left( {\frac{{a^{2} }}{j\omega } \mp \hat{\alpha }_{em} } \right)$$

18 $$\hat{\alpha }_{ee} = \frac{1}{\eta }\left( {\frac{{a^{2} }}{j\omega } \pm \hat{\alpha }_{em} } \right)$$

In omega structure chirality ${ }\alpha_{em}^{co}$ parameter is zero. By applying this condition in Eq. (12), the reflection coefficient can be written as shown in Eq. (19). By analyzing this relationship, one can easily perceive that by varying $\alpha_{em}^{cr}$ (omega coupling dyadic), the co-polarized reflection from the opposite side can be controlled, while maintaining total absorption from the original front side.

19 $$E_{r} = \pm { }\frac{2j\omega }{S} \cdot \alpha_{em}^{cr} \overline{\overline{I}}_{t} E_{inc}$$

The co- and cross-polarizability dyadic (electric, magnetic, electro-magneto, and magneto-electro) of the proposed unit cell are calculated and shown in section III of this paper. To enable omega coupling combining two or more conductive inclusions in a one-unit cell is suggested in^30^. Using such approach to design omega bianisotropic metasurfaces not only reduces the fabrication cost and complexities but also makes them compatible with planar technologies.

### Geometrical configuration

**Figure 1:**
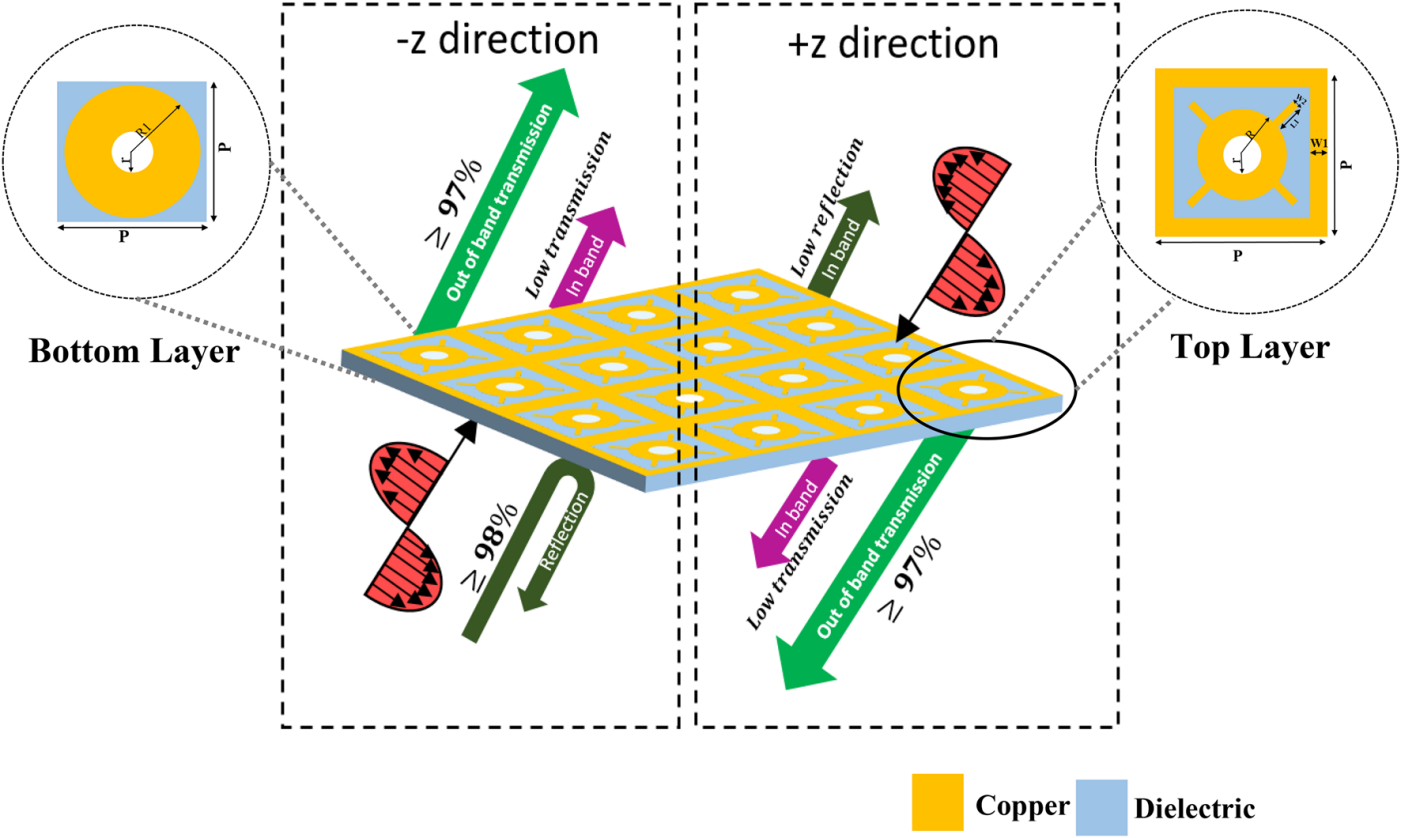
Schematic diagram of the omega bianisotropic metasurface along with geometric configuration of the top and bottom metallic layers of the unit cell. *The figure is created using Microsoft Power Point-Office 365-URL: https://www.office.com/powerpoint.

The proposed unit cell consists of two unique metallic inclusions imprinted on both sides of the dielectric slab, as shown in Fig. 1. These inclusions are designed on an inexpensive, flexible substrate (RO4350B Lo pro) with relative permittivity of 3.55, loss tangent of 0.0027, and thickness of 0.1016 mm. The upper side of the unit cell consists of a square patch enclosing a circle with four rectangular stubs, whereas the bottom layer consists of a simple circular patch. The hole is created to achieve maximum out-of-band transmission. The parametrically optimized dimensions are *p* = 7.5 mm, *t* = 0.1016 mm, *W*_1_ = 0.75 mm, *W*_2_ = 0.6 mm, *r* = 0.75 mm, *R* = 2.1 mm, *R*_1_ = 2.1 mm, *L*_1_ = 1.421 mm.

The presented structure is periodic in the x and y-axis, hence acts as an infinite planar array of electrically small resonant inclusion. These proposed bianisotropic unit cells are arranged in a square lattice with a period of “a” and excited by an arbitrary linearly polarized plane wave. It is worth mentioning here that the unit cell array period is kept smaller than the operating wavelength to avoid grating lobes in scattering spectra. The following key points are considered while designing the unit cell:

- The proposed structure should be geometrically asymmetric, so as to behave asymmetrically on excitation from the + z and –z- directions. Such asymmetry provides the required bianisotropic response in the structure.
- The proposed structure is designed using two conductive inclusions so that it enables omega coupling, and thus acting as an absorber and partially a reflective surface when excited from the + z and –z-axis respectively.
- Instead of the ground plane (conventionally used in absorbers), a unique circle with outer radius “R” and inner radius “r” is imprinted on the backside, consequently, increasing the out of band transmission. Furthermore, a hole is created to enhance this out-of-band transmission.

### Design evolution

**Figure 2:**
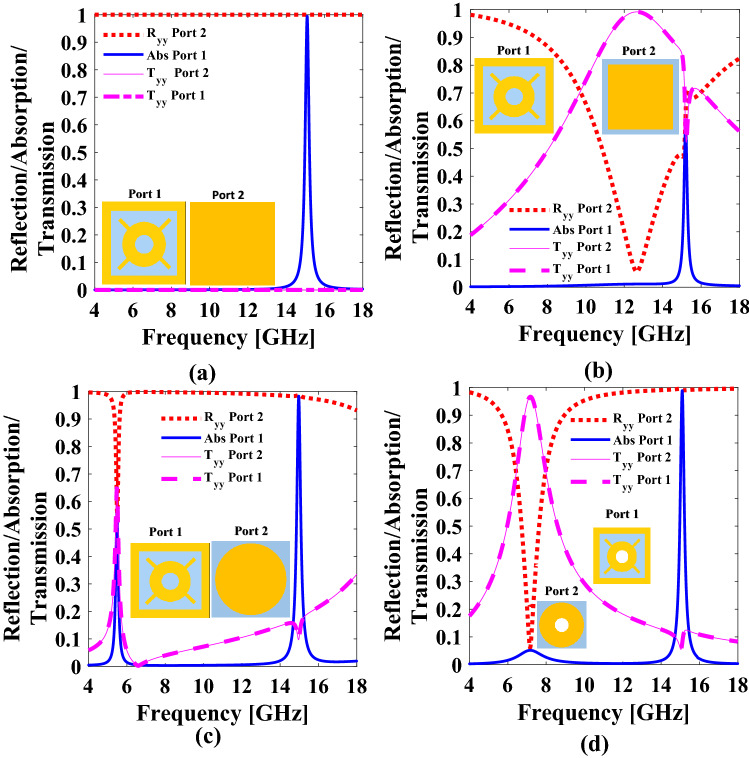
Unit cell design evolution (**a**) Square patch enclosing a circle with four rectangular stubs with a complete ground (**b**) Replacing a complete ground with a square patch (**c**) Replacing a complete ground with a circular patch (**d**) Creating a hole through the dielectric to enhance out of band transmission. *The figure is created using MATLAB ver. R2020A-URL: https://www.mathworks.com/products/matlab.html.

A bianisotropic metasurface is the one in which D(r,t) is anisotropically coupled to both E(r,t) and B(r,t). Similarly, H(r,t) is anisotropically coupled to both E(r,t) and B(r,t). In order to induce the omega coupling, the proposed structure is designed using asymmetric inclusions^30^, due to which it behaves asymmetrically on excitation from the + z and − z directions. The main purpose of this paper is to design a metasurface with asymmetric reflections providing maximum absorption when excited from port 1 and partial reflections when excited from port 2. The proposed design is optimized through systematic parametric analysis. The infinite unit cells along x- and y-axis are simulated in Computer Simulation Technology (CST) Studio Suite. The evolution steps of the proposed metasurface have been shown in Fig. 2a–d. These are few, but critical design steps performed to realize the final and optimized design. The resonance frequency of the proposed structure is calculated^37^ using Eqs. (20) and Eq. (21), i.e., 15.1 GHz.

20 $$f_{resonant} \cong \frac{{c_{o} }}{{2\left( {P + t} \right)\sqrt {\varepsilon_{re} } }}$$

where $c_{o}$ is the speed of light i.e. 3 × 10^8^ m/s and,

21 $$\varepsilon_{re} = \frac{{\varepsilon_{r} + 1}}{2} + \frac{{\varepsilon_{r} - 1}}{2}\frac{1}{{\sqrt {1 + 10\frac{t}{P}} }}$$

**Figure 3:**
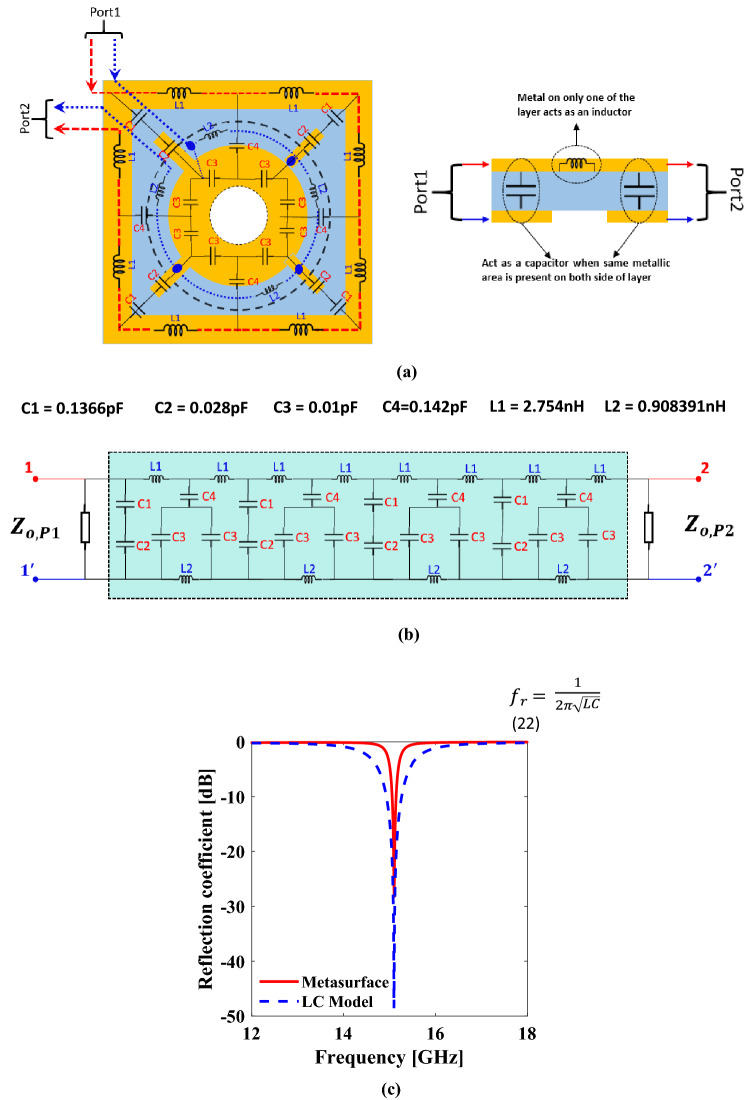
(**a**) Metasurface impedance model (**b**) Equivalent LC Circuit (**c**) Comparison of resonance achieved by LC model with metasurface. *The (**a**) and (**b**) are create using Microsoft Power Point-Office 365-URL: https://www.office.com/powerpoint; while the (**c**) is created using MATLAB ver. R2020A-URL: https://www.mathworks.com/products/matlab.html.

In step 1, the unit cell that consists of a square patch enclosing a circle with four rectangular stubs backed with the complete ground is designed as shown in ig. 2(a). In step 2, the ground plane is replaced by an optimized square patch to enable out-of-band transmission. As it can be seen from Fig. 2b the maximum out-of-band transmission is achieved at 13.1 GHz, while the absorption at 15.1 GHz is reduced to 50%. In order to maximize both the transmission and absorption from port 1 with partial reflection from port 2, a square patch is replaced with an optimized circular patch in step 3 as shown in Fig. 3c. As a result, maximum absorption of 99.46% from port 1 and partial reflections of 97% from port 2 is achieved at 15.1 GHz, where the out-of-band transmission reduces to 65%. In the final step, a hole of ‘r = 0.75 mm’ is created in a unit cell design to enhance the out of band transmission^39^ as shown in Fig. 2d. Hence, an absorption of 99.46% from port 1 and simultaneous partial reflections of 99% from port 2 at 15.1 GHz with maximum out of band transmission of 97% at 7.8 GHz is achieved.

### Unit cell equivalent model

To find the resonant frequency of the proposed structure, its LC circuit model is designed. To analyze the unit cell analogous to a transmission line, port 1 and port 2 are placed on the LC circuit model as shown in Fig. 3a. ne end of each of the two ports is connected to the upper side of the unit cell and the other end is connected to its lower side. Hence, the input signal entering port 1 and leaving port 2 experiences the same impedance as suffered by the overall metasurface. Starting from port1 to port 2, the conductive lengths, where no conductor is present on the opposite side, are modeled as an inductor, whereas the conductive lengths which have an equal metallic area on the opposite side are modeled as a capacitor. With this approach, the whole unit cell is modeled in terms of lumped elements, and its equivalent circuit is shown in Fig. 3b. The values of capacitors and inductors are calculated using parallel plate capacitance and flat wire inductance equations respectively^36^. This equivalent circuit is simulated using a circuit simulator namely Advanced Design System (ADS). From Fig. 3c, it can be deduced that the LC model and the metasurface resonate at a similar frequency i.e.15.1 GHz. Moreover, this resonance frequency can be tuned by tuning the capacitive and inductive area of the proposed unit cell using the following equation:

22 $$f_{r} = \frac{1}{{2\pi \sqrt {LC} }}$$

### Practical realization of metasurface based absorber via omega type bianisotropy

#### Polarizability dyadics analysis

**Figure 4:**
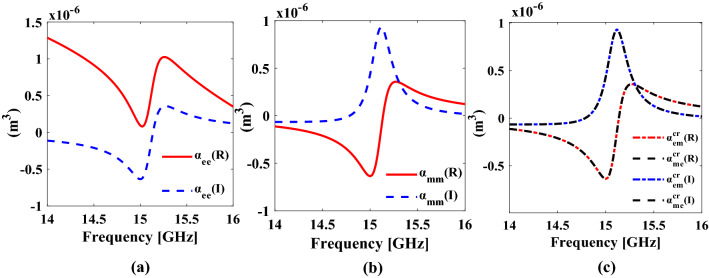
Individual Polarizability dyadics of designed omega bianisotropic (**a**) Electric polarizability (**b**) Magnetic polarizability (**c**) Electro-magneto and magneto-electric polarizability. *The figure is created using MATLAB ver. R2020A-URL: https://www.mathworks.com/products/matlab.html.

The polarizability dyadics of the proposed unit cell can be retrieved from its scattering parameters, proposed in^35^. The retrieved polarizability dyadics are shown in Fig. 4. Figure 4a, b show the co component of the electric ${\alpha }_{ee}^{co}$ and the magnetic ${\alpha }_{mm}^{co}$ polarizabilities respectively, and Fig. 4c shows the Electro-magneto and the magneto-electro response of the proposed unit cell. At the resonance frequency at 15.1 GHz, the real part of the electric polarizability does not cross zero, which is due to the non-zero static electric polarizability. In general, it can be concluded that the response is dominated by the induced electric dipole moment and therefore the mode is called electric mode^30^. It can be seen from Fig. 4c that both magnitude and phase of the cross-component of the electro-magneto (${\alpha }_{me}$) and magneto-electro (${\alpha }_{em}$) dyadics are equal hence the condition for omega coupling i.e. ${\alpha }_{em}^{cr}$ = ${\alpha }_{me}^{cr}$ is satisfied. It is due to the omega coupling, present at the resonance frequency, that the proposed metasurface acts as an absorber when illuminated from one side and, and like a partially reflecting surface PRS^31,32^ when illuminated from the other side.

#### Reflection/transmission coefficient

**Figure 5:**
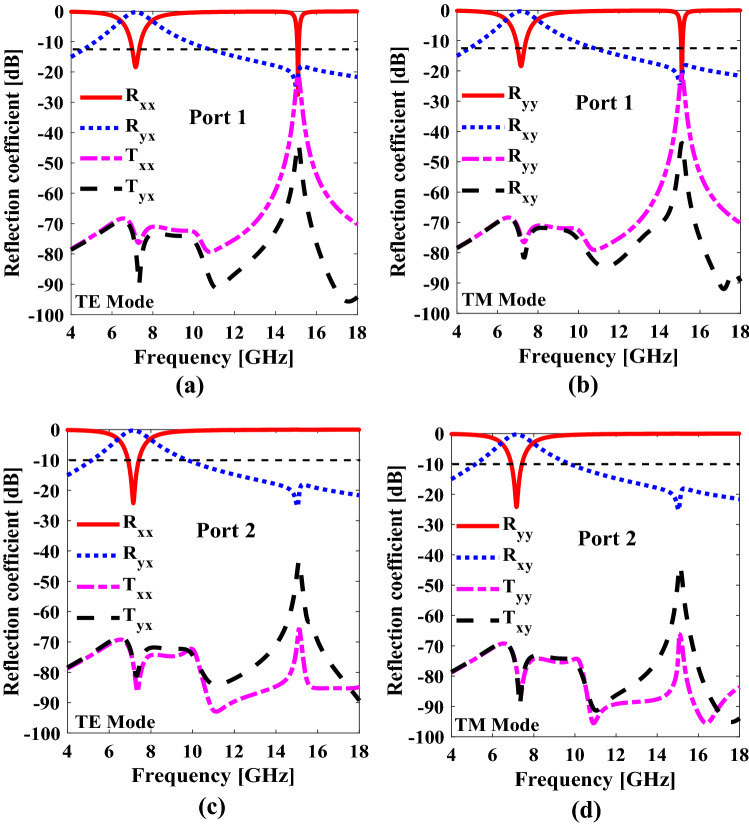
Transmission and reflection coefficients of proposed metasurface (**a**) TE mode for port 1 (**b**) TM mode for port 1 (**c**) TE mode for port 2 (**d**) TM mode for port 2. *The figure is created using MATLAB ver. R2020A-URL: https://www.mathworks.com/products/matlab.html.

For x polarized incident wave, the co and cross components of the reflected and transmitted wave are: $R_{xx} = \frac{{E_{rx} }}{{E_{ix} }}, R_{yx} = \frac{{E_{ry} }}{{E_{ix} }}, T_{xx} = \frac{{E_{tx} }}{{E_{ix} }} , T_{yx} = \frac{{E_{ty} }}{{E_{ix} }}$ and for y-polarized incident wave, the co and cross components of the reflected and transmitted waves are defined as:, $R_{yy} = \frac{{E_{ry} }}{{E_{iy} }}, R_{xy} = \frac{{E_{rx} }}{{E_{iy} }}, T_{yy} = \frac{{E_{ty} }}{{E_{iy} }} , T_{xy} = \frac{{E_{tx} }}{{E_{iy} }}$ . The aforementioned components are shown in Fig. 5a,b for port 1. Similarly, for port 2, the co- and cross- components of the reflected and transmitted wave, for both x and y polarized waves are shown in Fig. 5c,d. At the resonance frequency − 15.1 GHz- the values of the co-polarized reflection coefficient (port 1) for x and y polarized incident waves are approaching -25 dB and -22 dB respectively. The existence of such strong resonances is responsible for the nearly perfect absorption from port 1. However, for port 2, the co-polarized reflection coefficient ($R_{yy}$ and $R_{xx}$) remains high i.e., 99%, whereas all other reflected and transmitted components are below -10 dB. Therefore, it can be said that the proposed unit cell provides the asymmetric reflection at the resonance frequency when illuminated by EM waves from opposite sides.

#### Absorption

The absorption of the proposed metasurface for port 1 can be found using the following mathematical relationship^37,38^:

23 $$A\left( \omega \right) = 1 - \left| {R_{yy} } \right|^{2} - \left| {R_{xy} } \right|^{2} - \left| {T_{yy} } \right|^{2} - \left| {T_{xy} } \right|^{2}$$

where

24 $$\left| {R_{yy} } \right|^{2} = \left| {\frac{{Z_{in} - Z_{o} }}{{Z_{in} + Z_{o} }}} \right|^{2}$$

**Figure 6:**
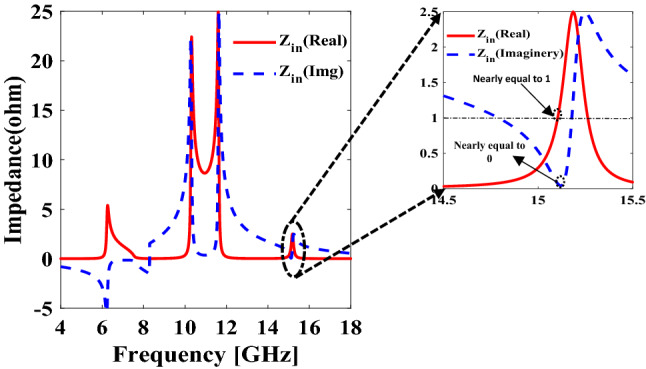
Effective impedance of metasurface. *The figure is created using MATLAB ver. R2020A-URL: https://www.mathworks.com/products/matlab.html.

Here $Z_{o}$ is the characteristic impedance of the free space that is equal to 377 Ω and $Z_{in}$ is the effective impedance. To calculate the value of the effective impedance ($Z_{in}$), constitutive parameters are extracted using susceptibilities^31,32^. Figure 6, it can be easily observed that the value of $Z_{in}$ is 0.955 i.e., 360 Ω. By putting these values in Eq. (15), $\left| {R_{yy} } \right|^{2}$ comes turns out to be 0.0005, and the extracted value of $\left| {T_{yy} } \right|^{2}$ is equal to 0.0018. By inserting the calculated values of $\left| {R_{yy} } \right|^{2}$ and $\left| {T_{yy} } \right|^{2}$ in Eq. (23) the absorption at 15.1 GHz becomes approximately equal to 99.7%.

**Figure 7:**
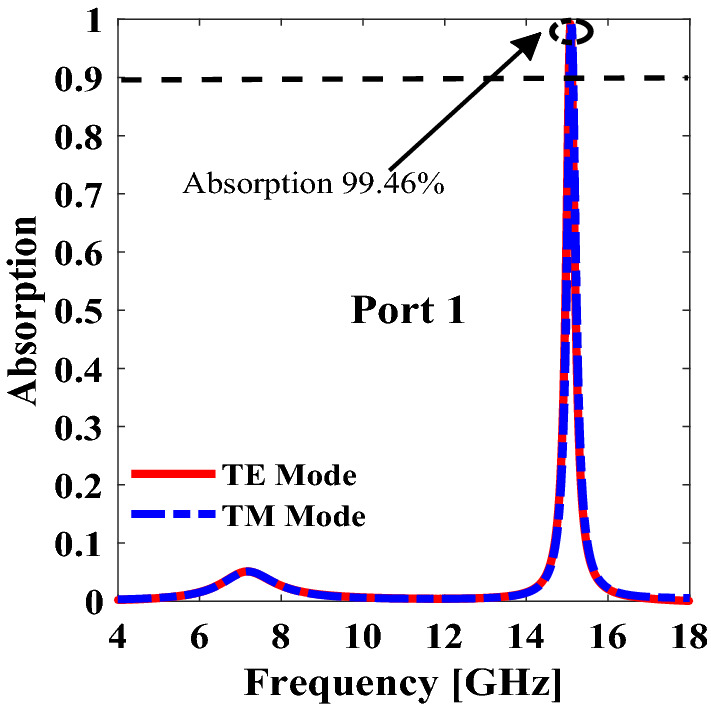
Absorption from port 1 for both x-polarized and y-polarized. *The figure is created using MATLAB ver. R2020A-URL: https://www.mathworks.com/products/matlab.html.

The absorption of port 1 for linearly polarized incident wave is calculated from the simulated results and it is shown in Fig. 7. It is evident that absorption of 99.46% is achieved at 15.1 GHz for both x and y polarized incident waves.

#### Out-of-band transmission

**Figure 8:**
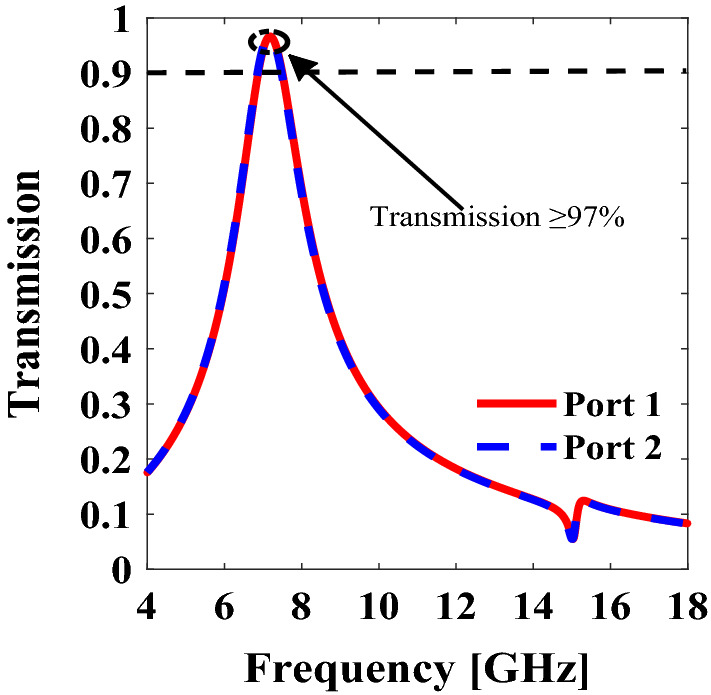
Out-of-band transmission from port 1 and port 2. *The figure is created using MATLAB ver. R2020A-URL: https://www.mathworks.com/products/matlab.html.

Unlike the traditional absorptive metasurfaces proposed earlier, the presented metasurface not only offers maximum in-band absorption, but it also gives maximum out-of-band transmission. In the proposed metasurface, a unique asymmetric geometry and a hole are used to enhance the out-of-band transmission. From Fig. 8, it can be verified that symmetric transmission of 97% is achieved at 7.8 GHz. This exclusive property makes this metasurface a suitable candidate for those applications, where the maximum in-band absorption with non-zero out-of-band transmission is required.

#### Angular stability

The robust response against oblique incidences plays a crucial role in many practical applications, thus, angular stability analysis is performed. An angularly stable metasurface, is a structure for which the response of the metasurface in normal incidence remains invariant when the same EM wave is incident at oblique angles. The major factors that contribute to angular stability are given below:

1. *Thickness* The electrical thickness of a substrate should be chosen in such a way that it should be smaller than the wavelength at the resonant frequency so that the spatial uniform fields can be induced in the substrate, which is immutable to variation of the angle of incidence^31,32^. Hence, Rogers4350B Lo pro substrate with a thickness of 0.1016 mm (electrical thickness of 0.005 λ at the resonant frequency) is chosen for the metasurface implementation.
2. *Unit cell size* The overall unit cell size (p=7.5 mm, 0.37 λ) is also kept smaller than the wavelength at the frequency of interest so that the uniform spatial fields may appear across the unit cell, which again makes this structure immutable to variations of the angle of incidence.
3. *Unique configuration* A unique geometric configuration with optimized dimensions is used so that it becomes angularly stable. To check the angular robustness of the proposed unit cell inclusion, the angle of incidence of EM wave is varied from 0˚ to 45˚.

**Figure 9:**
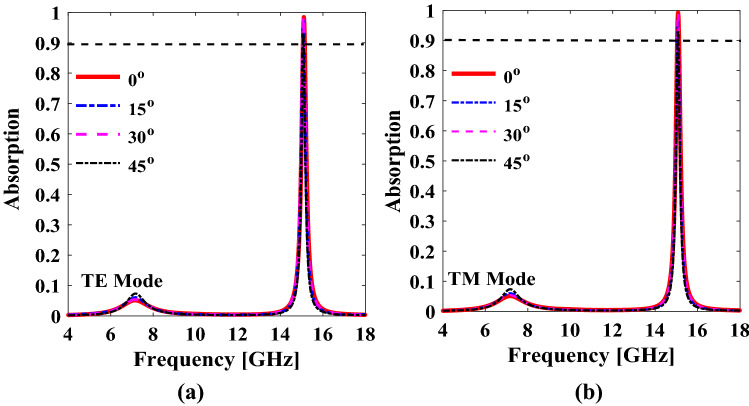
Incident angle increased from 0° to 45° (**a**) TE Mode (**b**) TM Mode. *The figure is created using MATLAB ver. R2020A-URL: https://www.mathworks.com/products/matlab.html.

It is evident from Fig. 9a,b that the response of the proposed unit cell is stable up to 45˚ for both x and y polarized incident waves.

#### Polarization insensitivity

**Figure 10:**
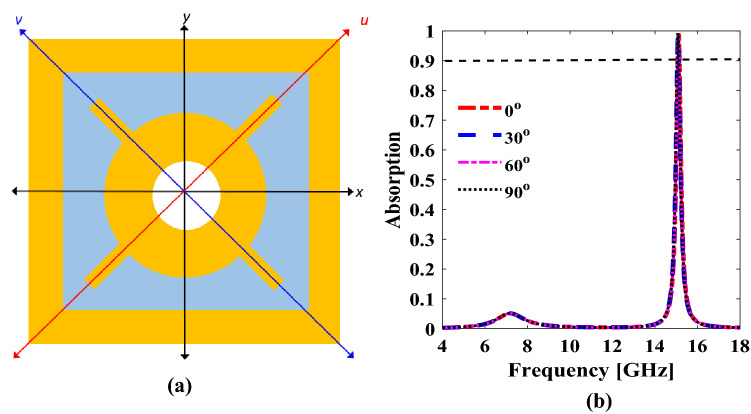
(**a**) 90º rotated symmetry (C4) and mirror symmetry along u-, v-, x- and y-axes (**b**) Metasurface response when electric field polarization is rotated from 0° to 90°. *The figure is created using MATLAB ver. R2020A-URL: https://www.mathworks.com/products/matlab.html.

Polarization sensitivity is also a desirable property for many practical applications. For the validation of this property for the proposed metasurface, the polarization of the incident wave is rotated from 0˚ (TE) to 90˚(TM). It can be easily noted from Fig. 10b that the absorptive response of the proposed metasurface remains immutable due to the cyclic-4 (C4) symmetry i.e., 90˚ rotated symmetry and mirror symmetry presented along x, y,u, and v axes as shown in Fig. 10a.

## Experimental results and discussion

**Figure 11:**
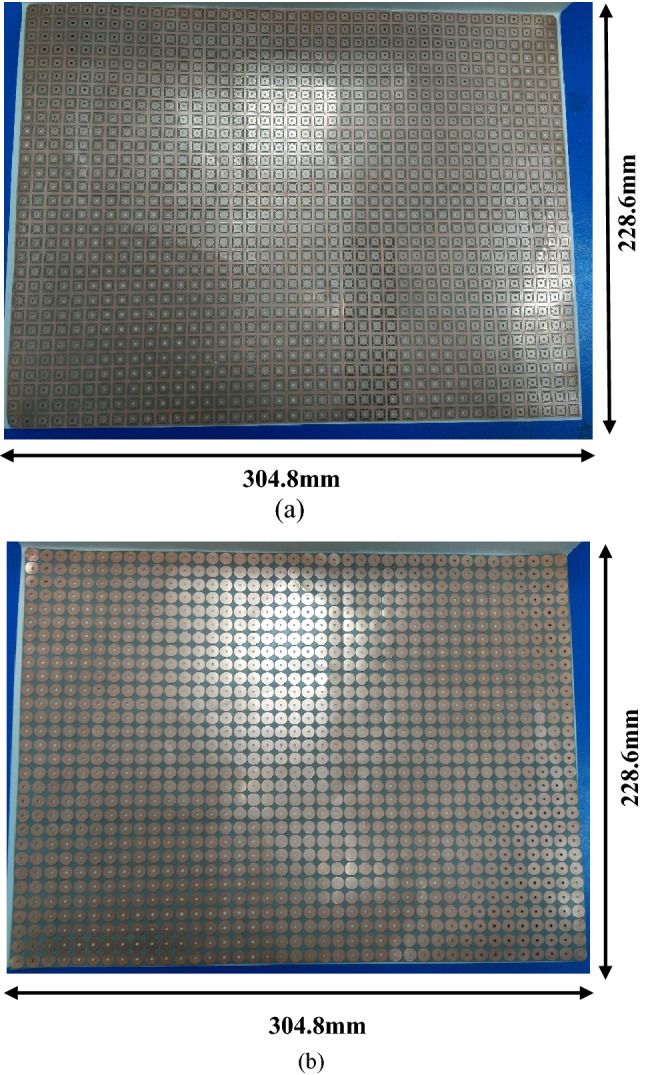
Fabricated prototype (**a**) Top layer (**b**) Bottom layer.

**Figure 12:**
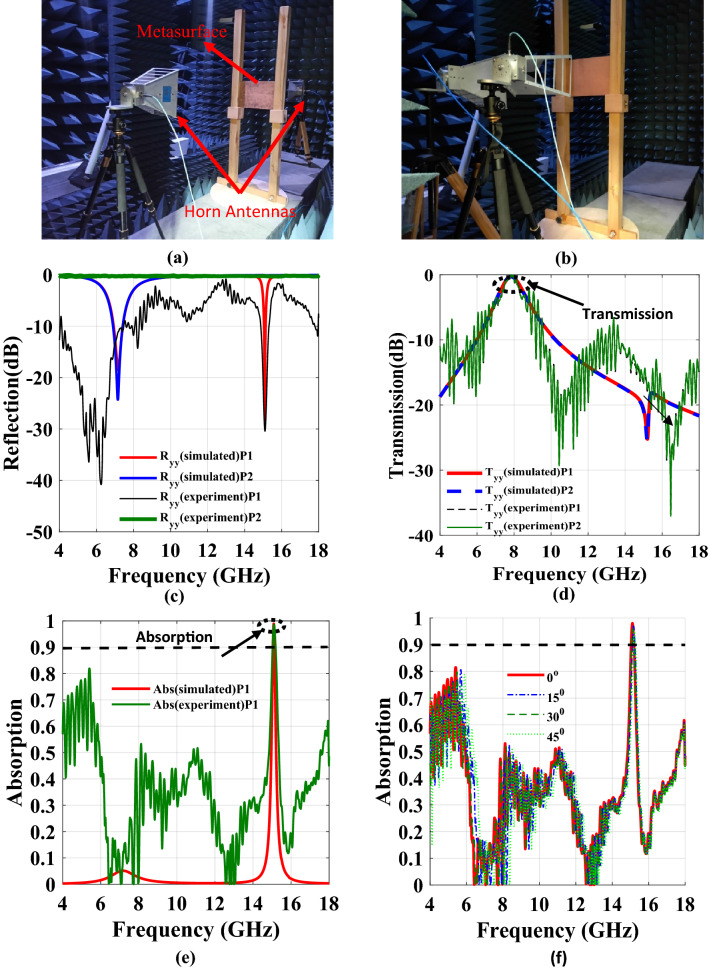
(**a**) Fabricated prototype of metasurface (**b**) Setup for the experimental measurements (**c**) Comparison between simulated and experimented reflections (**d**) Comparison between simulated and experimented transmissions (**e**) Comparison between simulated and experimented absorption (**f**) Experimented Angular Stability. *The Fig. 3c–f are created using MATLAB ver. R2020A-URL: https://www.mathworks.com/products/matlab.html.

To validate the performance of the proposed omega-type bianisotropic metasurface, a prototype was fabricated on Rogers’s 4350B Lo pro substrate having 0.1016 mm thickness. The fabricated PCB consists of 41 × 30-unit cells and its overall size is 304.8 × 228.6 mm^2^ as shown in Fig. 11a,b. The holes on the PCB are created using non-PTH drilling. An experimental setup is created inside an anechoic chamber where two horn antennas (operating frequency range of 2—18 GHz) are utilized for illuminating the surface and receiving the reflected/transmitted waves, as shown in Fig. 12a,b. These antennas were placed on the same side at a 120 cm (Fresnel region)^41,42^ from the surface for the reflection measurements. As far as the transmission measurements are concerned, the antennas are separated by a 240 cm distance and the metasurface is kept in the middle as shown in Fig. 12a^30^. As a reference for reflection and transmission measurements, the metallic plate and air measurements have been carried out before performing the actual prototype’s measurements. The horn antennas are attached to the vector network analyzer (Anritsu-MS46122B) through coaxial cables. To measure co-polarized transmission ($T_{yy}$ or $T_{xx}$), both transmitting and receiving antennas were placed either vertically (for $T_{yy}$) or horizontally (for $T_{xx}$). Similarly, for the measurement of the co-polarized reflection, the two antennas were placed parallel to each other as shown in Fig. 12b, for $T_{yy}$ both the transmitting and receiving antennas were vertical, while for $T_{xx}$ both antennas were placed horizontally.

It is worth mentioning that only co-components of transmission/reflections are considered in the measurements because the cross-components remained below -50 dB throughout the frequency band. The experimental results are summarized in Fig. 12c–e, and they are in reasonable agreement with the simulated results. It can be verified in Fig. 12c,d, that the phenomena of asymmetric reflection and symmetric out-of-band transmission are validated from the experimental measurements. The small discrepancies can be attributed to multiple reflections from the surrounding items, manual handling errors and the substrate tolerances. Moreover, the slight deviation is due to the finite size of the fabricated metasurface as opposed to the infinite size assumption that is made for the simulation model.

A comparison of the implemented omega bianisotropic metasurface with MSFs reported in literature is summarized in Table 1. The proposed flexible metasurface slightly improved absorption when compared with previously reported omega metasurfaces and it outperforms the competition in terms of out-of-band transmission (97%). At the same time, its normalized unit cell size is the smallest (0.37 λ) and the used material is flexible in nature contributing to the multiple functionalities of the proposed metasurface.

**Table 1:** Comparison with the state-of-the-art.

References	Max absorption (%)	PI (polarization insensitivity)	Flexible	Unit cell size	Angular stability	Out of band transmission (%)	Thickness [mm]
^30^	95	No	No	0.44 λ	40°	20	0.254
^31^	99	Yes	No	1.43 λ	**Up to 60° (< 6 GHz)**	< 10	2.4
**This work**	**99.46**	**Yes**	**Yes**	**0.37 λ**	**Up to 45°**	**97**	**0.1016**

## Conclusion

In this paper, a flexible omega bianisotropic metasurface has been presented. It is shown that the designed metasurface acts as an absorber (giving maximum absorption of 99.46%) when illuminated from port 1, whereas, on simultaneous illumination deriving from port 2 it behaves like a partially reflective surface. The proposed metasurface causes very high out-of-band transmission of up to 97%. The response of the presented metasurface remains the same for both TE and TM polarized waves or any arbitrary linearly polarized wave. Moreover, the response of the metasurface is stable for oblique incidences up to 45º. Such metasurfaces, with maximum absorption properties, partial reflections, and symmetric out-of-band transmission can be used in cavity antennas for gain enhancement with RCS reduction, for passband and stopband filtering applications, and due to its flexibility, it can be used in conformal antenna applications.

## Data Availability

The datasets generated during and/or analyzed during the current study are available from the corresponding author on reasonable request.
