# Author Correction: A multifunctional ultrathin flexible bianisotropic metasurface with miniaturized cell size

**DOI:** 10.1038/s41598-021-99946-x

**Published:** 2021-10-08

**Authors:** Tania Tamoor, Nosherwan Shoaib, Fahad Ahmed, Tayyab Hassan, Abdul Quddious, Symeon Nikolaou, Akram Alomainy, Muhammad Ali Imran, Qammer H. Abbasi

**Affiliations:** 1 National University of Sciences and Technology (NUST), Research Institute for Microwave and Millimeter-Wave Studies (RIMMS), Islamabad, 44000 Pakistan; 2 University of Cyprus, KIOS Research and Innovation Center of Excellence, Nicosia, 2109 Cyprus; 3 Frederick Research Center (FRC) and Frederick University, 1036 Nicosia, Cyprus; 4 Queen Mary University of London, School of Electronic Engineering and Computer Science, E1 4NS London, UK; 5 University of Glasgow, James Watt School of Engineering, Glasgow, G12 8QQ UK; 6 Artificial Intelligence Research Center (AIRC), Ajman University, Ajman, 346 UAE

Correction to: *Scientific Reports* 10.1038/s41598-021-97930-z, published online 16 September 2021.

The original version of this Article contained errors in Table 1, where the cited References were incorrectly given.

Incorrect:

**Table:** 

**References**
^26^
^27^
**This work**

Correct:

**Table:** 

**References**
^30^
^31^
**This work**

The original Article has been corrected.

